# Occupational therapy and return to work: a systematic literature review

**DOI:** 10.1186/1471-2458-11-615

**Published:** 2011-08-02

**Authors:** Huguette AM Désiron, Angelique de Rijk, Elke Van Hoof, Peter Donceel

**Affiliations:** 1 Department of Occupational, Environmental and Insurance Medicine, Katholieke Universiteit Leuven, Kapucijnenvoer 35, B3000 Leuven, Belgium; 2 Department of Social Medicine, Faculty of Health, Medicine and Life Sciences Maastricht University, P.O. Box 616, 6200 MD Maastricht, The Netherlands; 3 Department of Experimental and Applied Psychology (EXTO), Faculty of Psychological and Educational Science, Vrije Universiteit Brussel - Pleinlaan 2, B1050 Brussels, Belgium; 4 Belgian Cancer Centre, Scientific Institute of Public Health, Brussels, Belgium

## Abstract

**Background:**

The primary aim of this review study was to gather evidence on the effectiveness in terms of return to work (RTW) of occupational therapy interventions (OTIs) in rehabilitation patients with non-congenital disorders. A secondary aim was to be able to select the most efficient OTI.

**Methods:**

A systematic literature review of peer-reviewed papers was conducted using electronic databases (Cinahl, Cochrane Library, Ebsco, Medline (Pubmed), and PsycInfo). The search focussed on randomised controlled trials and cohort studies published in English from 1980 until September 2010. Scientific validity of the studies was assessed.

**Results:**

Starting from 1532 papers with pertinent titles, six studies met the quality criteria. Results show systematic reviewing of OTIs on RTW was challenging due to varying populations, different outcome measures, and poor descriptions of methodology. There is evidence that OTIs as part of rehabilitation programs, increase RTW rates, although the methodological evidence of most studies is weak.

**Conclusions:**

Analysis of the selected papers indicated that OTIs positively influence RTW; two studies described precisely what the content of their OTI was. In order to identify the added value of OTIs on RTW, studies with well-defined OT intervention protocols are necessary.

## Background

Restoring the ability to work is a key element in the rehabilitation of adult patients (aged 16-65 years). The primary goal of occupational therapy (OT), as part of the rehabilitation program, is to enable people to participate in the activities of everyday life including the ability to work. Occupational therapists achieve this outcome by working with people and communities to enhance their ability to engage in the "occupations' (used in terms of activities, and not only referring to employment) they want to, need to, or are expected to do. OT can involve, in order to reach the therapeutic goals, modifying the occupation itself or the environment [1]. According to the World Federation of Occupational Therapists (WFOT), the aims of Occupational Therapy (OT) are *"... to promote, develop, restore and maintain abilities needed to cope with daily activities to prevent dysfunction. Programs are designed to facilitate maximum use of function to meet demands of the person's working, social, personal and domestic environment... " *[1].

Assisting patients to return to their job is clearly an important part of the therapeutic effort of occupational therapists [2], the OT process is based on initial and repeated assessments in individual patients. Assessment includes the use of standardized procedures, interviews, observations in a variety of settings and consultation with significant people in the person's life. Functionality, the ability to perform activities in daily life, leisure and work and the possibility to participate in all aspects of life (including work) are part of the OT assessments. The results of these recurrent assessments form the basis of the therapeutic program plan, with inclusion of both short- and long-term aims of treatment. This plan must be relevant to the person's developmental stage, habits, roles, life-style preferences and the person's environment.

OT interventions, being part of the therapeutic plan, are designed to facilitate performance of everyday tasks and adaptation of settings in which the person works, lives and socializes. Interventions are directed towards developing, improving, and restoring daily living skills, work readiness, work performance, play skills, leisure capacities and enhancing educational performance skills (objectives) [3]. Re-assessments in different phases of the rehabilitation process are used to check results, and (re-)direct therapeutic goals.

Following Holmes [4], rehabilitation must focus on identifying and overcoming the health, personal/psychological, and social/occupational obstacles to recovery and (return to) work from this point of view, vocational rehabilitation reflects a wide variety of interventions, including meaningful occupations through voluntary work, sheltered work, supported employment and open employment opportunities. As a therapeutic intervention, return to work includes also patients who are assisted by their (occupational) therapists to regain access to the (premorbid) type of work.

From that point of view, vocational rehabilitation is one of the methods that can be put to use by OT on behalf of reaching the patients goals when RTW and/or regaining productivity (in a more large meaning) is at stake. In practice, vocational rehabilitation is realized through a partnership between the patient and all the rehab-team members, including OT. Especially for patients who suffer from symptoms that not only endanger their (labour-) participation while the rehabilitation process is on-going, but who risk being disabled on longer terms (because of permanent limitation of chronic problems), OT is assumed to be a relevant part of the whole rehabilitation program [5].

Since no evidence was found on behalf of breast-cancer survivors (specific population in which the researchers at first took interest), it was decided to enlarge the focus on RTW and OT for all patients confronted with long term effects of diagnose/treatment, including problems on RTW.

In the lecture of Whyte [6], held at the 57th John Stanley Coulter memorial lecture, the author stated that much discussion has been going on, on the need to enhance evidence base supporting rehabilitation practice. Within the professional group of occupational therapists, both researchers and practitioners indicate that - like Whyte points out in the conclusions of his lecture- they need to acknowledge that empirical work alone will not develop the science of rehabilitation. Therefore, attempting to add to the work that Whyte, Lee and others advocate, this review centralizes current evidence with regard to the added value of OT for patients aiming at return to work, regardless of the categories of patients to who this intervention was provided (RTW).

For different groups of patients, the importance of RTW is increasing both for personal and societal reasons [7]. Early RTW programs represent a bridge towards employment for an injured worker. Wright argued that OT practitioners are ideally suited to guide that transition [8]. Evolution in the medical treatment of different pathologies (e.g. cancer, AIDS) initiated evolutions in rehabilitation programs offered to patients. As part of the multidisciplinary rehabilitation effort, OT focuses on restoring activity and participation (including labour participation). Restoring and/or maintaining activities and participation of clients in different dimensions of life (self-maintenance, productivity and leisure) [9] is the main goal for occupational therapy interventions (OTIs). Occupational therapists should deliver evidence-based services in order to ensure quality in the input of OT within the whole rehabilitation program. Evidence supporting the effectiveness of OTIs in terms of RTW is particularly useful, as it can be used to develop specific programs targeting RTW.

Primary goal of this review was to assess the effect of OTIs on RTW and if effects are found, to describe what OT aspects contribute to the effects. Secondary aim was to select the most efficient OTI for an intervention to promote RTW. Subsequently, this review focussed on the following questions:

• What are the effects of OTIs in vocational rehabilitation on RTW?

• What aspects of OT contribute to these effects?

Evidence-based information not only will stimulate professionals in rehabilitation teams to optimize the quality of services these professionals provide (and more specifically the work of occupational therapists). It will also support the quality of patient outcomes in terms of preventing loss of income, decreasing the number of sick-leave days, and increasing quality of life [10].

## Methods

Five electronic databases (Cinahl, Cochrane Library, Ebsco, Medline (Pubmed), and PsycInfo) were used to search for pertinent articles published between 1980 and September 2010. The patient population/problem (P), intervention (I), comparison (C), and outcome (O), or PICO technique, was used to find relevant information and to formulate relevant questions that best match the capabilities of database search engines. Using the PICO elements as guideline, focus of this review could be rigorously maintained on patients suffering from an injury or illness that causes temporary incapacity to work and on patients participating in rehabilitation programs including OT.

### Identification of studies

**Figure 1:**
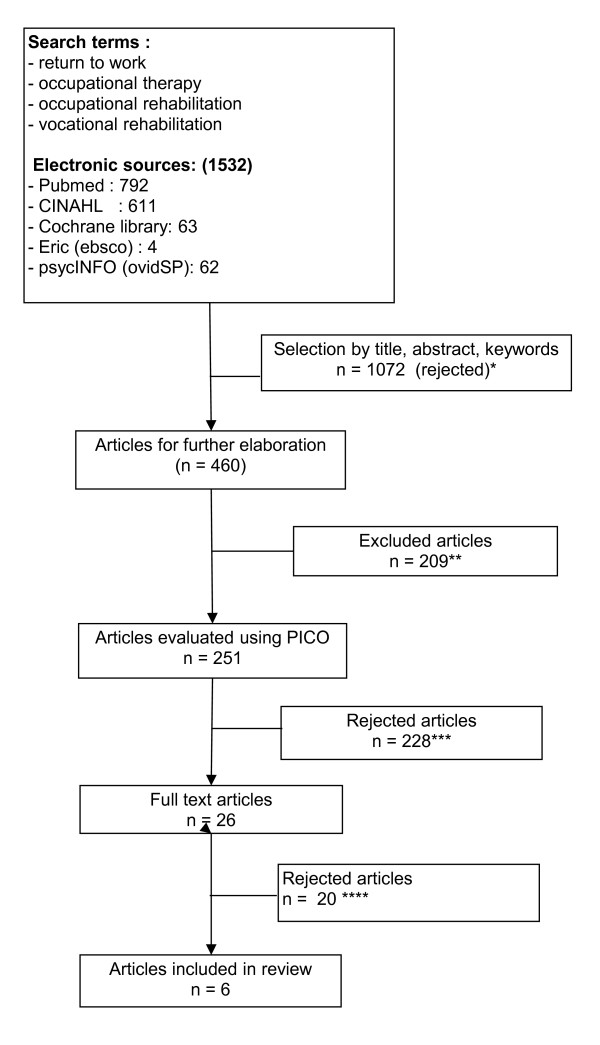
**Search strategy**. *: Criteria used to exclude selected studies. • Studies in which the term "occupational therapy" did not occur in title and/or abstract. • studies that did not contain "occupational therapy" and "return to work" in the title, abstract and/or key-words. • Studies in the field of "occupational medicine/occupational rehabilitation" that discussed return to work (RTW) but did not mention occupational therapy (OT). **: Exclusion-criteria: • No correct reference information mentioned (no authors name indicated, lists of articles from congress books,...). • Doubles (studies that appeared in multiple electronic sources). ***: After screening with PICO items, reviews and descriptive studies were excluded but intervention studies were included. ****: studies excluded after analysing the full text (role of OT in RTW process), excluding those studies that did not explicitly mention OT as a part of the multidisciplinary rehabilitation programme.

Figure 1 shows the selection process of articles for full-text analysis (n = 26). Inclusion criteria were:

a) The studies had to be either randomised controlled trials (RCTs) or cohort studies and written in English;

b) The participants had to be patients of working age (18-65 years) that had participated in a rehabilitation program;

c) The OTI had to be a part of a multidisciplinary rehabilitation program aiming at RTW, regardless of the patient population the intervention was provided for.;

d) The interventions examined had to be RTW multidisciplinary rehabilitation programs that included OT (i.e. the therapeutic efforts had to be part of a defined program whose specific goal was to help patients re-enter or remain in the work force);

e) The outcome measures had to measure work-related outcomes such as RTW, sick leave, or employment status;

f) Studies were published between 1980 till September 2010.

Vocational rehabilitation studies that did not explicitly describe or mention OT involvement were excluded (see Figure 1).

A preliminary pilot study used to determine how to direct the literature search, showed that OT was often part of multidisciplinary teamwork or program described under "vocational rehabilitation". Therefore, "vocational rehabilitation" was added to the search terms "occupational therapy" and "return to work" (see Figure 1).

By screening the titles, abstracts, and keywords for the terms "occupational therapy" and "return to work" potential studies were identified. Studies published in German [11-13] were also included. Additionally, using the names of researchers authoring relevant studies, a "snowball search" was used by screening the reference lists of selected articles for pertinent references. Additional research material suggested by OT experts was screened too.

### Data extraction

Data extraction from the included papers was performed by one researcher (H.D.) and checked for accuracy by the other authors. Disagreement in data extraction was resolved by consensus. As the structure and content of the OT programs remained vague, except for the study of Lambeek et al., the authors were invited by e-mail between October and December 2010 to provide more information. From 3 studies additional information was provided [14-16].

### Quality assessment

Quality assessment was performed by evaluating methodological quality of the studies [17-19]. Internal validity, study methodology, and external validity were assessed.

#### Internal validity

The criteria used to check internal validity were the quality of the sampling, the quality of randomisation and experimenter blinding, sufficient number of participants for statistical evidence, and description of confounders and bias. Three levels of scoring were used: (A) when the number of participants was sufficient to produce reasonably acceptable statistical power, randomisation was carried out carefully for RCTs (including blinding) and it was described whether (and in which way) confounders and bias were taken into account; (B) when all criteria were met as far as practically possible, but some compromises were necessary or when a description of how confounders and bias were treated was lacking; (C) all other cases.

#### Methodological quality

Criteria used for the methodological evaluation were appropriateness of data analysis, loss-to-follow-up/selective loss-to-follow-up, intention-to-treat analysis/per protocol analysis, and compliance. Three levels of scoring were used: (A) when all of these criteria were described in the article and treated appropriately; (B) when appropriate data analysis was carried out and some but not all of the criteria were described in the article or some comments could be made on the methodological approach; (C) when appropriate data analysis was carried out, but the methodology was not described or was poorly described.

#### External validity

Criteria used to evaluate external validity were whether the conclusions were applicable to situations in other geographic areas, importance (quantitative) of the populations for which the conclusions are likely to be applicable, and scope of generalizability (non-specificity of the sample). Three levels of scoring were used: (A) RCT studies that were likely to be applicable to large populations and likely to be geographically independent; (B) cohort studies that were likely to be generalizable and/or that examined somewhat specific populations; (C) studies that examined very specific populations (e.g. traumatic brain injury in military personnel on duty).

## Results

Evidence was gathered about the effect of OTIs in vocational rehabilitation on RTW by analysing peer-reviewed papers on OTIs that focussed on RTW. The search focussed on RCT and cohort studies, initially obtaining 1532 titles of potentially pertinent papers (see Figure 1). For the 251 articles that met the inclusion criteria, abstracts were analysed for the explicit use of OT as a part of the rehabilitation interventions aiming at RTW. Of these articles, 26 were selected for further full-text review. These papers were subjected to further analysis using the inclusion criteria mentioned. This review focussed on studies that specifically and explicitly focussed on OT as part of a multidisciplinary rehabilitation program. This strict inclusion criterion did lead to finally withhold six studies.

### Studies selected

Six papers [14-16,20-22] finally met the quality criteria. These six studies included 899 patients older than 18 years (active age) that participated in rehabilitation programs aimed at RTW. All studies included patients - suffering from differing problems - who had jobs at the time of the research project in which they participated and patients were supported by social security system and/or private insurances. The patients were evaluated after the program (1 week to 42 months after discharge). Three of the studies were RCTs; the remaining three were cohort studies. Methodological characteristics and quality are presented in Table 1.

**Table 1:** Study and patient characteristics and methodological evaluation

Author	Diagnosis	Design	Follow-up	Internal validity*	Methodology*	External validity*
Jousset et al., 2004	Chronic low-back pain	RCT/single blind	Evaluation 6 months after programme in rehabilitation centre	B	B	A

Joy et al., 2001	Low-back injury	Retrospective cohort study	Telephone interview 4 weeks after termination of treatment programme	B	B	C

Lambeek et al., 2010	Chronic low back pain	RCT	Assessment at baseline, 3,6,9,12 months	A	A	A

Schene et al., 2007	Major depressive disorder	RCT	Assessments at baseline, 3, 6, 12, and 42 months	B	B	A

Sullivan et al., 2006	Whiplash injury	Longitudinal cohort study	Structured interview questions 1 year post-protocol treatment	A	B	B

Vanderploeg et al., 2008	Traumatic brain injury (military personnel)	RCT intent-to-treat: 2 different treatments	Follow-up telephone calls 1, 6, 12, and 24 months after discharge	A	A	C

One of the selected studies was of high quality (A score) for all the items. Two selected studies showed good internal validity (score B), and 3 showed moderate internal validity (score C). Four studies were of moderate methodological quality (score C), and only two studies--Vanderploeg *et al. *[16] and Lambeek [14] --were of high quality (score A). Three studies--Jousset *et al. *[20], Schene *et al. *[15] and Lambeek *et al. *[14] and Vanderploeg *et al. *[16] did have limited external validity (score C) because they focussed on very specific target groups. Potential bias from selecting patients in insurance-paid programs was recognised by Vanderploeg et al.[16]and Lambeek *et al. *[14], but not specifically mentioned by the other studies.

Although all studies in this review focussed on RTW and the role of OT in that process, analysis showed many differences which hindered comparison of the studies and their results. All studies showed an effect on RTW in a program in which OT is involved, although large heterogeneity is found. Studies differed in type of intervention, patient type involved, operational definition of the notion RTW and in follow-up period.

### Outcome measures and definition of return to work

All of the selected studies denoted RTW as an outcome measure but their definition of RTW and what RTW involves varied widely (Table 2).

**Table 2:** Objectives, return to work (RTW) definitions and outcome measures

Author	Objective	Defining RTW result	Outcome measures
Jousset et al., 2004	Compare RTW (1) in patients participating in a multidisciplinary functional restoration programme to RTW in patients participating in active individual therapy	Significantly lower mean number of self-reported sick-leave days	• Number of self-reported sick-leave days during 2 previous years were noted at start of 5 week programme • Number of self-reported sick-leave days 6 months after the programme • RTW within 1 week after programme • Subjective rating: ➢ Ability to work ➢ Improved physical condition

Joy et al., 2001	RTW after work-hardening programme	Either part-time of full-time RTW at the time of follow-up phone calls (in original or alternative job)	• Functional capacity • Age • Length of injury (days) • Time in program (days) • Work status (did or did not RTW) • Pain level • Pain tolerance (% improvement) • Activity tolerance (% improvement)

Lambeek et al., 2010	to evaluate the effectiveness of an integrated care programme, combining a patient directed and a workplace directed intervention, for patients with chronic low back pain	Duration of sick leave due to low back pain in calendar days from the day op randomisation until full RTW in own or other work with equal earnings for at least four weeks without recurrence, partial of full.	• Primary outcome: duration of time off work (work disability) • Secondary outcome: ➢ intensity of pain and functional status ➢ the integrated care programme substantially reduced disability due to chronic low back pain in private and working life ➢ improvement of pain between groups did not differ significantly

Schene et al., 2007	Work resumption	Significant difference between TAU(4) and TAU + OT (5) in time between baseline assessment and time of RTW for patients who did not work at baseline assessment Total hours worked during each 6-month period up to 42 months for the total population	• Depression • Work resumption • Work stress • Service use and qualitative evaluation • Economic evaluation

Sullivan et al., 2006	Compared percentage of RTW in patients participating in PGAP + PT (6) to those participating in PT (7) alone	Returning to full-time pre-injury employment or alternative employment	• RTW (primary outcome variable) • Catastrophizing • Fear of movement or reinjury • Perceived disability • Pain severity

Vanderploeg et al., 2008	Comparing RTW or return to school in patients participating in 2 rehabilitation approaches	Current status of paid employment or school enrolment (either full- or part-time, not as part of a sheltered workshop)	• RTW/school • Living independently • Satisfied with life • Chance in martial state since injury • Social withdrawal • Worrying • Depressed mood • Irritability • Angry behaviour

Legend Objectives, return to work (RTW) definitions and outcome measures

1. RTW:return to work; 2 FRP: functional restoration program; 3 AIT: active individual program; 4 TAU: treatment as usual; 5 TAU + OT: treatment as usual + occupational therapy; 6 PGAP: Progressive goal attainment program + physical therapy; 7 PT(physical therapy); 8 TBI (traumatic brain injury)

Concluding whether a given intervention has (successful) effects regarding its goal (RTW) depends on the definition of "successful RTW" (see table 2). In the studies reviewed here, both the definition of successful RTW, which ranged from part-time to full-time employment, and the time of follow-up, which ranged from 1 week to 42 months, differed substantially. In the study of Joy *et al. *[21], successful RTW was measured in terms of the percentage of RTW compared to the situation before participants entered the program. Although the other selected studies also compared different forms of treatment including OT, they did not demonstrate precisely how each professional discipline contributed to RTW.

### What are the effects of OTIs in vocational rehabilitation on RTW?

All OTIs affected RTW. Jousset *et al. *[20] found significantly lower mean numbers of self-reported sick-leave days in the functional restoration group who took part in OT. Joy *et al. *[21] suggested that multidisciplinary programs (including OT) such as work-hardening and functional restoration may be of benefit in helping the patients identify and resolve issues that often contribute to disability exaggeration leading to greater RTW success independent of any changes in a patient's overall pain level. This parallels the findings of Lambeek *et al. *[14], who also concluded that disability decreased although improvement of pain did not differ between both groups. Schene *et al. *[15] found that adding OT to the usual treatment increased and accelerated work resumption of people suffering from depression. OT, however, did not accelerate recovery from depression. Results of the work of Sullivan *et al. *[22] revealed that a risk-factor-targeted intervention administered by physical therapists and occupational therapists can have a meaningful impact on RTW following whiplash injuries. The impact of their program was most pronounced for the subgroup of subjects who scored in the risk range on all psychosocial variables targeted by the program. Vanderploeg *et al. *[16] added (measured by in-person evaluations and structured telephone interviews at 1 year after the programs) the amount of help that the participating patients with traumatic brain injury received and details on any vocational activity over the year since completing the study protocol to their RTW measure. Their study found no difference in RTW between patients that received cognitive didactic and those who received functional experiential approaches during traumatic brain injury rehabilitation.

### What aspects of OT contribute to these effects?

Effects of OTIs in rehabilitation programs regarding RTW are recognisable, but large differences in settings, design, in- and exclusion criteria, disciplines concerned in the study-programs, and in outcomes made it difficult to determine the extent to which OT contributions to these interventions affected RTW (see table 3). The different OTIs, integrated in the multidisciplinary intervention are:

**Table 3:** Intervention description, OT elements in the intervention, assessment instruments and general conclusions

Author	Description intervention	OT elements in the intervention	Instruments used for assessments	General conclusions
Jousset et al., 2004	**Functional restoration programme (FRP) **including intensive physical training, occupational therapy, psychological support and dietic advices a day, 5 days a week, 5 weeks. **Active individual therapy (AIT): **1 hour treatment sessions, 3 times a week during 5 weeks (programme of exercises to perform alone at home for 50 min. on the 2 remaining weekdays.	Daily for 1.15 hrs. • Flexibility, • Endurance, • Co-ordination, • Weight lifting, • Work simulation	• Trunk flexibility by fingertip-floor distance • Trunk strength by isometric contraction (ITO et al & Biering-Sorensen) • Lifting: Progressive ISO-inertial lifting evaluation (PILE) • Level op pain: VASQoL & functional indexes • French version of Dallas pain questionnaire • Quebec back pain disability scale • Hospital anxiety depression scale • Use of prescript medication	FRP was more efficient then AIT in reducing the number of sick leave days, improving physical condition: • FRP from 102,3 to 28 days • AIT 109,8 to 48 days

Joy et al., 2001	**Work hardening programme:** • Job-specific work simulations • Physical conditioning • Education Patients who did RTW after work hardening program to patients who did not RTW after work hardening program	• Initial intake evaluation, • Daily activities schedules, • Case-management, • Pain management techniques, • Individual work simulation activities, • Discharge planning	• Study specific questionnairepain drawing (indicating where pain was felt) • 10 point pain level indication scale • Physical assessment • Functional abilities testing for 16 physical demands • Exit-questionnaire • Improvement scale (pain tolerance, activity tolerance) at exit program • Determining RTW by contacting patients after discharge (1, 6, 12 and 24 months)	No significant differences due to age, gender, length of injury, days spent in work hardening program or change in pain level Significant difference in pain tolerance (men: 26,8% vs 42,0%; women: 24,2% vs 39,1%) No significant difference in activity tolerance

Lambeek et al. (2010)	**Care as usual** • medical specialist • occupational physician • general practitioner • and/or allied health professionals **Integrated care** • coordination by clinical occupational physician • team members: • medical specialist, • OT, • physiotherapist; • integrated care protocol: • care-management by occupational physician (from 1 to full sustainable work or to week 12) • work place intervention (using occupational therapist brainstorm (from week 3 tot week 12) • graded activity (from week 2 till1 full sustainable work or to max. week 12)	• Assessment patients functional capacity at baseline • Workplace intervention • 26 sessions of graded activity	• Questionnaires at baseline and 3,6,9,12 months • primary outcome (full RTW): - Self reported sick leave - Data from dbase of the occupational health service • Secondary outcome: - VAS (pain) - Roland disability questionnaire (functional status) • Prognostic factors for duration of sick leave - Job content questionnaire (potential work related psycho-social factors) -Dutch musculoskeletal questionnaire (data on workload)	The integrated care programme substantially reduced disability due to chronic low back pain in private and working life

Schene et al., 2007	**Treatment as usual (TAU**) (out-patient psychiatric treatment for depression) • Clinical management • antidepressants • 30 min visits every 2-3 weeks compared to **TAU + Occupational Therapy (OT**)	• **Diagnostic phase (4 weeks) **: five contacts with a detailed occupational history, video observation in a role -played work situation, contact with an occupational physician of the patients employer and a plan for work reintegration • **Therapeutic phase (24 wee**ks): 24 weekly group sessions and 12 individual sessions3 sub phases: preparation to work reintegration, contacting the place of work and if possible starting to workin individual sessions: further analysis of the relationship between work and depression, exploration of work problems, support and evaluation of work resumption • **Follow-up phase (20 weeks) **: three individual visits	• DSM-IV (major depression Episode) • Beck Depression Inventory (BDI) • Questionnaire organisation stress (QOS) • Study specific questionnaires	The addition of OT did not accelerate recovery from depression The addition of OT accelerates and increases work resumption The addition of OT did not increase work stress

Sullivan et al., 2006	Compare RWT rates of **additionally Progressive goal attainment programme (PGAP) **to the results of a historical cohort enrolled in a functional restoration physical therapy intervention.	• Education and reassurance • Maintaining activity log • Activities scheduling • Walking programme • Increasing activity involvement • Overcoming psychological obstacles to activity involvement	McGill pain questionnaire, pain rating index (MPQ) Pain catastrophizing scale (PCS) Tampa scale for kinesiophobia(TSK) Pain disability Index (PDI)	A psychosocial risk factor targeted intervention in combination with physical therapy can lead to significant increases in the probability of RTW following whiplash injuries. (75% vs 50%) The combination of psychosocial intervention with physical therapy may emerge as a viable and cost-effective approach for the prevention of prolonged pain and disability following musculoskeletal injury.

Vanderploeg et al., 2008	**Cognitive-didactic programme (CD)**: 1,5 to 2,5 hours of protocol specific cognitive-didactic interventions (Individual treatment) with another 2 to 2,5 h daily of OT & physiotherapy Emphasis on building self-awareness No real life tasks and settings **Functional experiential rehab therapy (FE) **1,5 to 2,5 hrs of protocol specific functional-experimental treatment with another 2 to 2,5 h daily of OT & physiotherapy. Focus on developing useful functional abilities or skills	**All** Basic activities of daily living, range of motion, mobility **CD**: Training 4 cognitive domains (attention, memory executive functions, pragmatic communication) Trial and error approach **FE**: Real life performance situations and common tasks Learning by doing	Functional Independence Measure (FIM) Disability Rating Scale (DRS) present state examapathy evaluation scaleneurobehavioral rating scalelife satisfaction (self-rating and clinical interview)	No difference between cognitive-didactic and functional-experiential approaches to TBI rehab on primary 1 year global outcome measures. However, patients at the cognitive treatment arm had better post treatment cognitive performance. At 1 year post injury, the overall rates of independent living and employment and/or student status were 58,9% and 37,2% respectively.

• Jousset et al. mention "work simulation" as part of the Functional Restoration Program but do not specify what exactly the content of that part of the program was, in which settings it was performed or what the specific approach of the occupational therapist was [20].

• Lambeek *et al. *[14] mention the contribution of occupational therapists in the description of the study, but in the specifications of different types of therapeutic services, provided in primary and secondary care, the description "occupational therapist" is not used. Nevertheless, the additional description of the protocol of the "integrated care " used in the study, point outs very clearly in what way occupational therapy was used. The main part of the work of the occupational therapist included in the study of Lambeek *et al. *[14] is to provide a workplace intervention. Being a member of the multidisciplinary team, the OT takes part in gathering patient information. In the additional report [23], a detailed OT protocol is included and supported by an "occupational therapist flow chart", thus indicating the OTI time-span. Every four weeks (telephone) conferences with the clinical occupational physician, physical therapist and medical specialist need to take place. The protocol mentions the issues that need to be discussed and the timeframe for the OTI.

• The content of a therapeutic program item like "work hardening" is mentioned in the work of Joy et al., but is not clear what the therapeutic actions are, what type of approach is used, what activities are performed [21].

• The program of Schene *et al. *[15] provides three individuals visits in the last phase. The information, separately published in the intervention protocol, clarifies precisely the content of the program [24].

• The OT part of the program of Sullivan et al contains "increasing activity involvement" but it is not specified in the study-report what precisely the therapeutic actions of the occupational therapist were [22].

• Vanderploeg *et al. *[16] mention that the OT was part of the multi-disciplinary team, but do not give further details on the content of the input of OT. The additionally provided information [25] gives more specific information on the research protocol, however, no specification of the precise content of the occupational therapy part of the program was indicated [25]. The protocol clarifies how the whole team had to collaborate but does not offer a detailed description of the specific actions of each discipline involved.

Whether OT had a meaningful role in the outcome of the different programs, is not only a result of the OT contribution itself, but also of the composition of the services offered by the multidisciplinary team concerned in the program (see tables 3 and 4).

**Table 4:** Study design, settings, in- & exclusion criteria, disciplines concerned and key measures/variables

Author	Design	Settings	In (I)- and exclusion (E) criteria	Disciplines concerned in multi-disciplinary team	Key measures/variables
Jousset et al., 2004	RCT/single blind	Patients of 3 counties in the west of France, referred to the multidisciplinary Low Back Pain clinic by industrial physicians, family doctors, specialists or social insurance medical advisers and assessed by a physiatrist, an occupational medicine specialist, a psychologist and an ergonomist	**I **: 18 - 50 years old, living in 3 counties in west of France, engaged in a non-limited contract, threatened, at risk of unable to work in their job situation by Low back pain LBP, not relieved by conventional medical or surgical intervention **E**: lack of motivation, major psychiatric diseases; no disabling (LBP), LBP of specific origin, recent surgery, cardiac of respiratory abnormalities after exercises stress, receiving disability pension, refusal to randomisation	• Aerobics, • Strengthening exercises, • Proprioception • endurance training by physiotherapist • OT • Balneotherapy • Psychologist • Dietic advice	• RTW after 6 months end program • Mean number of sick leave days • Physical criteria • Treatment appreciation • Intensity of pain • Quality of life • Functional indexes • Psychological characteristics • Number of contacts with medical system • Drug intake

Joy et al., 2001	Retrospective cohort study	Northern Californian work hardening program, patients authorised to attend by their workers compensation board	**I **: records from patients with low-back injuries referred to a work hardening program in Northern California from march 1989 to august 1996; at referral off work for 2 months or more since injury or surgery, entitled to workers- compen-sation benefits**E**: data from patients referred for reasons other than low back injury	• Physiotherapist • OT • Vocational counsellor • Psychologist • Workroom foreman	• Functional capacity • Age • Length of injury (days) • Time in program (days) • Work status (did or did not RTW) • Pain level • Pain tolerance (% improvement) • Activity tolerance (% improvement)

Lambeek et al. 2010	RCT	Primary care in the Netherlands 10 physiotherapy practices, one occupational health service, one occupational therapy practice Secondary care 5 hospitals in the Netherlands.	**I: **age 18 - 65; low back pain (for more than 12 weeks); visited outpatient clinic in participating hospitals; in paid work (self-employed and paid employed) for at least 8 hours/weekabsent (total or partial) from work **E**: patients absent from work >2 yearsworked temporally or for an employment agency without detachment; specific low back pain due to infection, tumour, osteoporosis, RA, fracture, inflammatory process; undergone surgery or invasive examinations within 3 monthsserious psychiatric or cardiovascular illnesswere pregnant; were engaged in a lawsuit against their employer	• Clinical occupational physician • Medical specialist • OT • Physiotherapist	• Primary RTW:duration of sick leave due to low back pain in calendar days from the day of randomisation until full RTW (or work with equal earnings for al least 4 weeks without recurrence, partial or full). • Secondary pain (3,6,12 months) functional status (3,6,12 months)

Schene et al., 2007	RCT	research was conducted as part of the Programme for Mood Disorders of the Department of Psychiatry of the Academic Medical Centre of Amsterdam	**I**: age above 18; major depressive disordersingle episode of recurrent without psychotic features; no history of psychosis, manic, hypo manic or cyclothymic features; no history of active drug or alcohol abuse or dependencea Beck Depression Inventory scale of > 15 work reduction of at least 50% of regular hours worked per week because of depression (with a minimum of 10 weeks and a maximum of 2 years) **E**: after telephonic screening on inclusion criteria, patients received a regular psychiatric evaluation(2 visits) by two trained senior psychiatrists who checked again for the inclusion criteria	• Psychiatrist (trained for the program) • OT	• Age • Gender • Married or not • Living alone or not • Education (< high school or not) • Employment before illness (hours/week) • Major depressive disorderBeck Depression Inventory (BDI) • Questionnaire Organisation Stress (QOS) • Study specific questionnaires (qualitative data)

Sullivan et al., 2006	Longitudinal cohort study	5 eastern Canadian rehab centres (10 week standardized psychosocial intervention program, secondary prevention)	**I **: whiplash injury following an vehicle accident (grade I and II), score within the risk range (i.e. above 50 percentile) on at least one of the psychosocial measures targeted in the program, patient in one of 5 rehab clinics in eastern Canada whose staff had attended a 2-days training workshop on PGAP intervention techniques, being employed prior to their motor vehicle accident, providing informed consent participating in a functional restoration physical therapy program **E**: not being employed	• Physical therapist • OT • Occupational health nurse • Office assistant (interviews)	• RTW (primary outcome variable) • Catastrophizing • Fear of movement or reinjury • Perceived disability • Pain severity

Vanderploeg et al., 2008	RCT intent-to-treat: 2 different treatments	CARF standards of care interdisciplinary rehabilitation services in 4 veteran administration cure inpatient TBI rehabilitation programs (USA).	**I **: moderate to severe Traumatic Brain Injury (TBI) within preceding 6 months (Glasgow outcome scale) and/or focal cerebral contusion (CT or MRI), RLAS cognitive level of 5 to 7 at time of randomisation, 18 years or older, active duty military member or veterananticipated length of needed TBI rehab of 30 days or more **E**: history of prior inpatient acute rehab for the current TBI, history of a prior moderate to severe TBI or other pre-injury severe neurological or psychiatric condition	• physical therapy • OT • Speech therapy • Neuropsychological therapy	• RTW/school • Living independently • FIM • DRS • Satisfied with life • Chance in martial state since injury • Social withdrawal • Worrying • Depressed mood • Irritability • Angry behaviour

Remarkable in the selected studies was the mixture of terms used to describe multidisciplinary teams. The disciplines concerned in "multidisciplinary rehabilitation" across the six studies show that a great variety of disciplines is involved in RTW programs. Moreover, many of the authors failed to differentiate between occupational therapy, physiotherapy, and physical therapy. Lambeek *et al. *[14] however, describe precisely the contribution of each discipline in the multidisciplinary team, including a flow chart of the process in which each of those team members was involved in the integrated care protocol.

Recognizing the role that OT plays in the overall therapeutic effort (by using the WFOT definition of the profession [1]), is not obvious, but for experienced OT's nevertheless very well recognisable in the papers by Jousset [20], Joy, Sullivan [22] and Vanderploeg [16,25]. This finding supports the statement of Lee and Kielhofner that specific evidence of OTIs is lacking.

Schene et al.'s [15] conclusions show that a holistic approach (e.g. psychosocial intervention combined with physical therapy) is useful for preventing loss of capacities (and thereby loss of the ability to work). Moreover, Sullivan *et al. *[22] suggested that a holistic approach can increase successful RTW by 25%. Vanderploeg *et al. *[16,25] determined the contribution of OT (cognitive-didactic versus functional-experiential approaches) during different stages of the therapeutic process. Referring to the definition of OT used in this review, the cognitive-didactic approach can be considered as the OTI in the Vanderploeg et al. study [16]. Although they did not find significant differences after one year of rehabilitation, they found that participants in the cognitive-didactic program showed better post-treatment cognitive performance.

## Discussion

The primary aim of this systematic review was to identity the effectiveness in terms of Return to Work (RTW) of Occupational Therapy Interventions (OTIs) in rehabilitation patients with non-congenital disorders. In general, findings show results in favour of using OT in a multidisciplinary rehabilitation when targeting RTW. The effect of OT, measured at follow-up in terms of the number of sick-leave days or in terms of employment status, showed good results.

A great deal of the literature (1027 of 1532 articles) fitted the search terms but did not examine interventions that specifically and explicitly included OT. The selected literature (1532 preliminary results) contained a lot of descriptive studies, qualitative research and reviews. Therefore, using a strict set of inclusion criteria, the search was focussed on RCTs or cohort studies, leaving descriptive literature aside. As a result, this review is based on six studies and reveals that better RTW results are achieved when rehabilitation focuses on functionality using OT, as already suggested by the WFOT and confirming the reasoning of Wright.

Since a large variety of interventions, with different patient-populations were performed by the occupational therapists of these programs used in the studies, it was difficult to compare - and thereby generalise - the results of these studies. In order to do so, both uniform terminology and specific, detailed descriptions of the therapeutic content of the OTIs would be needed. This supports the statement of Lee and Kielhofner [2], as they point out the lack of well-described definitions in the field of OT research. Research efforts indicate that performing or simulating patients' "work activity" during rehabilitation can be very valuable in assisting them to restore their labour-participation [26-28]. Therefore, it would be very beneficial for constructing "good practice" to determine in further research efforts exactly what sort of interventions an OT program needs to implement in order to be as successful as possible, as provided in the work of Lambeek *et al. *[23] and Schene *et al. *[15].

Schene *et al. *[15] demonstrated that, in comparison to a psychosocial intervention alone, adding OT increases RTW for people suffering from major depression. Results of Lambeek *et al. *[23] tend to support this statement regarding RTW for patients suffering from low back pain. Jousset *et al. *[20] showed a decreased number of sick-leave days in workers with low-back injuries.

There are thus indications that OT is a key element in the therapeutic program. Nevertheless, the scientific evidence on which these OTIs would be based, can only - to ensure solid evidence - be retrieved from two studies. In literature, occupational therapists report many challenges in adopting and implementing evidence-based principles to practice. According to Lee and Kielhofner, research indicates that current OT practice is still not strongly grounded in theory, occupation and evidence [2]. They state that, although occupational therapists provide a range of work-related interventions, specific evidence related to OT in the area of vocational rehabilitation remains somewhat limited [2].

Lee and Kielhofner found that published works tend to focus on issues of scholarship rather than implications for practice, thereby often limiting the practical implementation of the findings into OT practice. Nevertheless, Lee and Kielhofner also state that experiences (of some authors of projects in which occupational therapists are involved) indicate that simultaneous consideration of theory and evidence is advantageous to achieve occupation-focuses best practice [2].

Simultaneous addition of other interventions on the other hand, such as care management and physical therapy (graded activity) [18], clog the precise effect of the OTI. In their report, Lambeek *et al. *[14,23] do not comment on a possible cross-over or a mutual re-enforcing effect of components of the integrated care program as effected by respectively the clinical occupational physician, the physical therapy and the OT. They do, however, in the discussion part of their report, regret the fact that the study design was not suitable for assessing the effectiveness of the individual components of the integrated care intervention (integrated care management, workplace intervention, and graded activity). In this study the randomization compared usual care to a workplace intervention, in which the medical team was enlarged by the employer, aiming at identifying the barriers and coming up with solutions. Average patient contact for providers was the same for the occupational medicine physician and the occupational therapist with approximately 17 sessions with the physical therapist. Clearly, an unambiguous identification of the OTI was not possible. The study indicates the fact that OT can/does have a role to play when RTW is at stake. Lambeek *et al. *[14] presume that a factorial design, and additional qualitative research focussing on the experience of healthcare professionals and patients, could give more insight into the effective components of the intervention.

In the protocol used in Schene *et al. *[15], the authors specify the socio-economical context in which the intervention took place. Referring to the Dutch legislation and the care that can be provided within that legal framework, de Vries and Schene [24] clarify how "care as usual" needs to be understood; this information was lacking in other studies.

In occupational therapy literature, different authors have been mentioning the importance of "work" as a life-domain that cannot be neglected by the therapeutic programs offered by occupational therapists [29-34]. Gibson and Strong [35] stated that occupational therapists can play a major role in work rehabilitation for RTW by assessing and rehabilitating workers with a disability injury. Kinébanian & Le Granze indicate that Lee and Kielhofner [2] described and synthesised evidence about work-related OTIs the Model of Human Occupation (MOHO) [36]. MOHO-based work programs have been shown to have positive impact in improving vocational outcomes for a broad range of clients, including persons with chronic illness [2,7,9,12,13,37,37-40]. Studies included in the work of Lee and Kielhofner [2] point to the need for further research to more fully examine the effectiveness of programmes involving different diagnostic groups.

The authors of this review agree with Lee and Kielhofner [2] that occupational therapists should put more effort in clearly documenting the specific therapeutic actions they deliver in the RTW process, as provided by Schene *et al. *[24] and Lambeek *et al. *[14,23]. Therapeutic actions such as work hardening, work simulation, preparation for work reintegration, contacting the place of work, starting work in individual sessions, exploration of work problems, support and evaluation of work resumption need to be described more precisely in order to document the specific content of OT actions and to be able to repeat these actions. When efforts of occupational therapists are described and taken into a precise protocol, taking the work of Schene et al. and Lambeek et al. as inspiration [14,15,23], comparison is facilitated.

As occupational therapists try to restore the abilities of their patients during the rehabilitation process, they need well-constructed evidence pertinent to the unique situations they may encounter. This supports both the occupational therapist and the patient to construct a therapeutic pathway that fits the unique and individual reality of the patient.

By pointing out both the base evidence for "good practice" and the need to construct valid and reliable OTIs, this review sheds light on how occupational therapists need to work in order to develop adequate therapeutic answers for patients' needs. As this systematic review is set up as a part of a research project, aiming on a RCT on OT and RTW, we also try to assist in overcoming the indicated shortcomings. Far too long, OT's have focused on practice "in the field", without publishing practical- or research results on their work. As - following the evolution the input of the WFOT - in more and more countries, not only bachelor-level research is done, but OT's are participating in research on master of PhD level, one can expect that more research (both qualitative and quantitative) will be published.

In the systematic search we carried out, the aim was to identify studies in which OT was involved, trying to find indication for further research. Except for the study of Vanderploeg *et al. *[16] no other publication could be found in which OT was separately measurable. This indicates the need for a (relatively young) profession like OT to clarify the effects that OTI can have in strengthening the work of the team and delivering benefit for patients on specific issues (function, activity and participation) in lives domains like self-care, leisure and productivity.

Using uniform terminology will clarify the existing confusion that stems from the use of different terms and content (e.g., occupational therapy versus physical therapy; return to work versus work resumption versus job re-entry). Eliminating this confusion can help caregivers and patients to get a clear notice of what service they can claim when an occupational therapist is included and what results they may expect when an OTI. Finally, in order to clarify and construct evidence supporting the value of OT in restoring labour participation for rehabilitation patients, much research still needs to be done.

## Conclusions

The goal of this systematic review was to analyse the effectiveness in terms of Return to Work (RTW) of Occupational Therapy Interventions (OTIs), in order to construct evidence for OTIs programs providing RTW assistance for rehabilitation patients. Descriptive literature and information from experienced practitioners in the field of OT reveal that occupational therapists are increasingly involved in assisting patients in restoring their workability. This systematic review provides sufficient evidence that rehabilitation programs that included OTIs do contribute to RTW, but it is not clear yet what the effective ingredients are, except for work place interventions [14]. Only six studies met the inclusion criteria and varied regarding population, outcome measure, or had weak descriptions of the methodology used. Thus, a univocal indication of "good practice" of an OTI aiming at RTW is lacking. Even though, the results of this review contribute to clarifying what steps need to be taken to construct the evidence needed and, even more, can stimulate occupational therapists and researchers in their efforts to continue the work that needs to be done.

## Competing interests

The authors declare that they have no competing interests.

## Authors' contributions

All authors were involved in the process of setting up the strategy for this study. PD supervised the study. HD carried out the research work itself, the other authors screened retrieved papers on in- and exclusion criteria and they also appraised the quality of the retrieved studies. The draft of the manuscript was supervised by PD, AdR & EvH.

All authors read and approved the final manuscript.

## Pre-publication history

The pre-publication history for this paper can be accessed here:

http://www.biomedcentral.com/1471-2458/11/615/prepub
